# Macrophage activation syndrome in children with Kikuchi-Fujimoto disease

**DOI:** 10.1186/s12969-023-00788-w

**Published:** 2023-01-25

**Authors:** Zixuan Shen, Jiayun Ling, Xiaona Zhu, Jun Yang, Tingyan He

**Affiliations:** Department of Rheumatology and Immunology, Shenzhen Children’s Hospital, 7019 Yitian Road, Shenzhen, 518038 China

**Keywords:** Kikuchi-Fujimoto disease, Histiocytic necrotizing lymphadenitis, Systemic juvenile idiopathic arthritis, Macrophage activation syndrome

## Abstract

**Background:**

Kikuchi-Fujimoto disease (KFD) is typically a benign, self-limiting inflammatory disease. However, some patients may have a prolonged or recurrent disease course, or present with life-threatening complications such as macrophage activation syndrome (MAS). In this study, we aimed to describe the incidence and clinical features of MAS in KFD and to access potential laboratory markers for the diagnosis of KFD-associated MAS.

**Methods:**

Patients with KFD were retrospectively enrolled from January 2015 to November 2021 at Shenzhen Children’s Hospital. Clinical data were collected from inpatient or outpatient medical records. Data collected included clinical manifestations, laboratory and imaging findings, treatment, and clinical outcomes. Data were analyzed using GraphPad Prism 8.0 statistical software (GraphPad Software Inc., La Jolla, CA, USA). A receiver operating characteristic (ROC) curve analysis was further performed to access the potential predictors for the KFD-MAS diagnosis.

**Results:**

Of 58 patients with a histological diagnosis of KFD, 15 (25.9%) patients had MAS. Compared to patients without MAS, patients with KFD-MAS presented with a higher proportion of skin rash (26.7%, *p *= 0.01), glucocorticoid treatment (80%, *p *= 0.003), and disease recurrence (33.3%, *p *= 0.04). KFD-MAS patients had lower absolute peripheral white blood cell (WBC, *p *= 0.02), platelet (*p *= 0.002), serum albumin levels (*p *= 0.01), and lymphocyte count (*p* < 0.0001), and higher lactate dehydrogenase (LDH) levels (*p* < 0.0001). ROC curve analysis showed that the cutoff values of absolute lymphocyte count, an absolute platelet count, serum albumin level, and serum LDH level for KFD-MAS diagnosis were < 1235/μL, < 171 × 10^6^/μL, < 35.6 g/L, and > 679 IU/mL, respectively.

**Conclusions:**

The presence of KFD-MAS in children may be more common than previously expected, especially in those with skin rash. KFD-MAS may be associated with a higher recurrence rate. An extremely elevated serum LDH level and moderate to severe lymphopenia may be useful diagnostic markers for MAS in KFD.

**Trial registration:**

Not applicable; this was a retrospective study.

**Supplementary Information:**

The online version contains supplementary material available at 10.1186/s12969-023-00788-w.

## Background

Kikuchi-Fujimoto disease (KFD), also known as histiocytic necrotizing lymphadenitis, is characterized by fever and cervical lymphadenopathy of unknown etiology. Systemic clinical manifestations may include night sweats, weight loss, fatigue, generalized lymphadenopathy, headaches, arthralgia, sore throat, rash, nausea, vomiting, and even neurological involvement [1]. As the symptoms and laboratory findings are non-specific, differential diagnoses include infectious lymphadenitis, malignancies such as lymphoma, and autoimmune diseases such as systemic lupus erythematosus (SLE) and juvenile idiopathic arthritis (JIA). Histopathological diagnosis by a lymph node biopsy is crucial to differentiate KFD from other etiologies.

KFD is typically a benign, self-limiting inflammatory process that resolves with supportive measures within months without any specific treatment. However, some patients may have a prolonged or recurrent disease course, or present with severe complications such as hemophagocytic Lymphohistiocytosis(HLH) [2–4]. HLH is a life-threatening syndrome of cytokine storm, characterized by hemophagocytosis, intractable fever, hepatosplenomegaly, cytopenia, hypertriglyceridemia, and hypofibrinogenemia. Serum proinflammatory cytokines, such as interferon-γ(or CXCL9 and CXCL10, chemokines induced by interferon-γ), interleukin-6, interleukin-10, and soluble interleukin-2 receptor alpha, a marker of T-cell activation, are usually excessively produced [5, 6]. Secondary HLH occurs in the setting of a malignant, infectious or autoimmune stimulus. Macrophage activation syndrome (MAS) is the term used to describe HLH that develops secondary to rheumatological diseases such as lupus and juvenile idiopathic arthritis, among others [7, 8]. The presence of MAS in adult KFD patients may be associated with adverse clinical outcomes including higher steroid usage and worse hospitalization outcomes, including a much higher rate of intensive care unit admission or in-hospital mortality [9]. The incidence and clinical features of MAS in KFD are not well understood in children. The potential markers for the diagnosis of MAS in children with KFD are still not well clarified.

Here, we performed a single, retrospective study to describe the incidence and clinical characteristics of MAS in children with KFD and to evaluate potential laboratory markers for the diagnosis of KFD-associated MAS (KFD-MAS).

## Patients and methods

### Study population and design

Patients with KFD were retrospectively enrolled from January 2015 to November 2021 at Shenzhen Children’s Hospital. The study was approved by the ethics committee of the hospital. Written informed consent was obtained from all patients’ legal guardians. All patients with KFD were confirmed based on a typical histological diagnosis. Exclusion criteria included patients with a possible alternative diagnosis such as autoimmune diseases (systemic lupus erythematosus, Sjogren's disease, etc.), leukemia, lymphoma, lymphoproliferative diseases, or active infection. Patients with other possible etiologies for HLH were not included either. Recurrence of KFD was defined as a diagnosis of KFD again after complete clinical remission over six months under immune-related drug withdrawal.

MAS was diagnosed according to the 2016 EULAR/ACR/PRINTO classification criteria, including fever, a ferritin level of ≥ 684 ng/mL, and fulfilling more than 2 of the following 4 criteria: platelet count ≤ 181,000/mL, aspartate aminotransferase (AST) level > 48 units/L, triglyceride level > 156 mg/dL, and fibrinogen level ≤ 360 mg/dL. KFD-associated MAS (KFD-MAS) was defined to fulfill the classification criteria for MAS and the diagnosis of KFD. Patients having MAS on admission and those who developed MAS within two weeks after admission were grouped as the KFD-MAS group.

## Data collection

Clinical data were collected from inpatient or outpatient medical records. Data collected included clinical manifestations, laboratory and imaging findings, treatment, and clinical outcomes. The clinical symptoms and laboratory data were collected at the onset of KFD. The clinical manifestations were listed in Table 1. Laboratory variables included total leukocyte, neutrophils, lymphocyte and platelet counts, erythrocyte sedimentation rate (ESR), levels of hemoglobulin, C-reactive protein (CRP), aminotransferases, albumin, ferritin, fibrinogen, lactate dehydrogenase (LDH), and auto-antibodies such as anti-nuclear antibodies (ANA). Management included glucocorticoids, intravenous immunoglobulin (IVIG), cyclosporine, etoposide, antibiotics, and other immunosuppressants.

**Table 1:** Clinical characteristics and management of KFD patients

	Without MAS (*n* = 43)	With MAS (*n* = 15)	*p*-value
**Demorgraphic Data**			
Age, years	8.5 (6.8–11.8)	10.58 (7.2–12.9)	0.86
Male to female ratio	1.69 (27:16)	2.75 (11:4)	0.54
Follow-up (years)	3.58 (2.33–5.25)	4.33 (3.0–5.5)	0.18
**Clinical manifestations**			
Fever	39 (90.7)	15 (100)	0.34
Hepatomegaly	14 (32.6)	5 (33.3)	> 0.9999
Splenomegaly	7 (16.3)	1 (6.7)	0.44
Skin rash	1 (2.3)	4 (26.7)	**0.01**
Arthralgia/arthritis	1 (2.3)	1 (6.7)	> 0.9999
Gastrointestinal symptoms	4 (9.3)	1 (6.7)	> 0.9999
Respiratory symptoms	4 (9.3)	3 (20.0)	0.36
Central nervous symptoms	7 (16.3)	2 (13.3)	> 0.9999

Data expressed as median (interquartile range) or n (%). Variables such as age and follow-up were acessed by Unpaired Welch’s t-test. Others were compared by Man-Whitney U test. *KFD* Kikuchi-Fujimoto disease, *MAS* macrophage activation syndrome

## Statistical analysis

Continuous variables were presented as medians with interquartile ranges (IQR), and categorical variables were presented as frequencies and percentages. Unpaired Welch’s t-test for continuous data and Man-Whitney U test for categorical variables were performed. Variables were compared at the onset of KFD in patients who developed MAS within two weeks of KFD versus those who did not develop MAS. We subsequently performed a receiver operating characteristic (ROC) curve analysis for the data with statistical differences between the two groups. Analysis was performed with GraphPad Prism 8.0 statistical software (GraphPad Software Inc., La Jolla, CA, USA). A p-value of < 0.05 was considered statistically significant.

## Results

### The clinical characteristics of KFD patients

We identified 58 patients with a histological diagnosis of KFD in Shenzhen Children’s Hospital, including 15 patients with KFD-MAS (15/58, 25.9%). Clinical characteristics were summarized and compared between KFD patients without MAS and those with MAS in Table 1 and Additional file 1. The proportion of skin rash was significantly higher in the KFD-MAS group (*p* = 0.01). Four patients in the KFD-MAS presented with scattered erythematous macules, and only one had plantar erythema in patients without MAS. Most other clinical features were not significantly different between these two groups, including fever, arthralgia/arthritis, hepatosplenomegaly, evidence of acute infection, and other clinical manifestations (Table 1).

### Comparison of the laboratory findings between KFD patients without MAS and those with MAS

Compared to KFD patients without MAS, the KFD-MAS group had significantly lower absolute peripheral white blood cell (WBC, *p* = 0.02), platelet (*p *= 0.002), serum albumin levels (*p* = 0.01), and lymphocyte count (p < 0.0001), and higher lactate dehydrogenase (LDH) levels (*p* < 0.0001). Serum ferritin, aspartate, and aminotransferase (AST) tended to be higher in the KFD-MAS group but were not significantly different between the two groups. There were no significant differences between the two groups in other laboratory data, including C-reactive protein (CRP), erythrocyte sedimentation rate (ESR), triglyceride, fibrinogen, hemophagocytosis by bone marrow smear, and autoantibodies (Table 2).

**Table 2:** Laboratory findings of KFD patients

	Without MAS (*n* = 43)	With MAS (*n* = 15)	*p*-value
Laboratory variables			
WBC count (/μL)	3940 (2990–4620)	2330 (1560–4200)	**0.02**
Hemoglobin (g/dL)	117 (109–127)	105 (97–129)	0.26
Neutrophil count (/μL)	1680 (1250–2500)	1230 (550–2900)	0.25
Lymophocyte count (/μL)	1540 (1280–1940)	1070 (800–1220)	** < 0.0001**
Platelet count (× 10^6^/μL)	213 (180–274)	161 (117–203)	**0.002**
AST (IU/L)	34.0 (25.0–46.0)	73.0 (49.0–96.0)	0.09
ALT (IU/L)	18.0 (11.0–32.0)	29.0 (13.0–55.0)	0.22
Albumin (g/L)	38.9 (37.1–40.2)	34.7 (32.6–38.5)	**0.01**
Ferritin (ng/mL)	207.6 (119.9–350.4)	1756.0 (785.2–3118.0)	0.11
Triglyceride (mg/dl)	1.2 (1.0–1.5)	0.96 (0.85–1.5)	0.77
Fibrinogen (mg/dL)	3.8 (3.4–4.3)	3.5 (2.6–4.5)	0.16
CRP (mg/L)	5.0 (2.1–11.9)	8.5 (1.3–24.3)	0.18
ESR (mm/h)	35.0 (24.0–47.0)	43.0 (32.0–50.0)	0.4
LDH (IU/mL)	380.0 (284.0–458.0)	857.0 (762.0–1005.0)	** < 0.0001**
Hemophagocytosis by bone marrow biopsy	16 (61.5, *n* = 26)	10 (71.4, *n* = 14)	0.73
Positive autobodies	9 (20.9)	2 (13.3)	0.71

Data expressed as median (interquartile range) or n (%). Variables such as positive autobodies and hemophagocytosis by bone marrow biopsy were acessed by Man-Whitney U test. Others were analyzed by Unpaired Welch’s t-test. *KFD* Kikuchi-Fujimoto disease, *MAS* macrophage activation syndrome, *WBC* white blood cell, *ESR* erythrocyte sedimentation rate, *CRP* C-reactive protein, *ALT* alanine aminotransferase, *LDH* lactate dehydrogenase, *AST* aspartate aminotransferase, *ANA* anti-nuclear antibodies

### Treatment and prognosis

Compared to KFD patients without MAS, patients with KFD-MAS had a significantly higher proportion of glucocorticoid treatment (*p* = 0.003) and KFD recurrence (*p* = 0.04). There were no significant differences between two the groups in other treatments or outcomes, including intravenous immunoglobulin (IVIG), cyclosporine, etoposide, tofacitinib, and progression to autoimmune diseases (Table 3).

**Table 3:** Management and prognosis of KFD patients

	Without MAS (*n* = 43)	With MAS (*n* = 15)	*p*-value
Treatment			
IVIG	7 (16.3%)	3(20.0%)	> 0.9999
Glucocorticoids	15 (34.9%)	12 (80.0%)	0.003
Cyclosporine	0 (0.0%)	1 (6.7%)	0.26
VP-16	0 (0.0%)	1 (6.7%)	0.26
Tofacitinib	0 (0.0%)	1 (6.7%)	0.26
Antibiotics	19 (66.7%)	10 (66.7%)	0.23
**Hosptial durations (days)**	11 (9–13)	11 (10–21)	0.41
**Recurrence of KFD**	4 (9.3%)	5 (33.3%)	0.04
**Progression to autoimmune diseases**	2 (4.7%)	1 (6.7%)	> 0.9999

*KFD* Kikuchi-Fujimoto disease, *MAS* macrophage activation syndrome, *IVIG* intravenous immunogloblin; VP16, etoposide; GCs, glucocortic

### Potential predictors for the KFD-MAS diagnosis

**Fig. 1:**
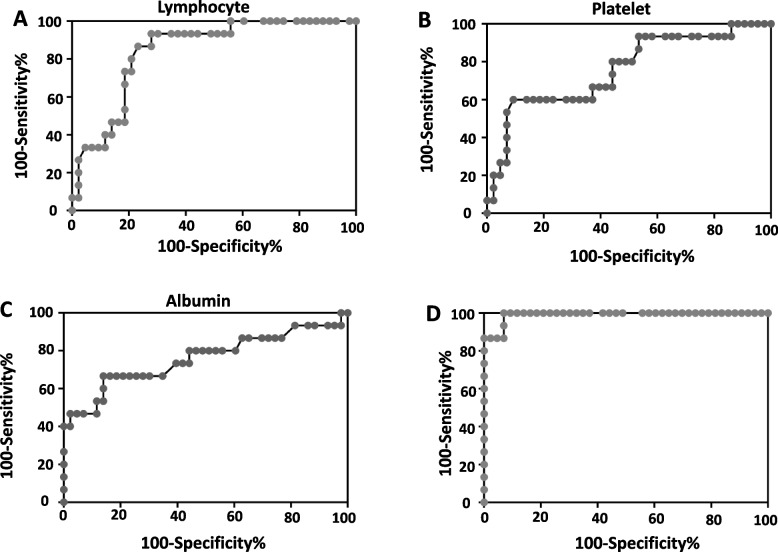
Receiver operating characteristic curves of laboratory markers

A ROC curve analysis was performed to access the potential laboratory markers for the KFD-MAS diagnosis. The results showed that an absolute lymphocyte count < 1235/μL (0.84, 95%CI: 0.74–0.95, *p* < 0.0001) distinguished the KFD-MAS group from the KFD without MAS group with 80.0% sensitivity and 79.1% specificity (Fig. 1 A). The cutoff values of an absolute platelet count, serum albumin level, and serum LDH level for KFD-MAS diagnosis were < 171 × 10^6^/μL (ROC-AUC 0.76, 95%CI: 0.61–0.90, *p* = 0.003) (Fig. 1 B), < 35.6 g/L (ROC-AUC 0.76, 95%CI: 0.59–0.92, *p* = 0.01) (Fig. 1 C), and > 679 IU/mL (ROC-AUC 0.99, 95%CI: 0.97–1.0, *p* < 0.0001) (Fig. 1 D), respectively. From ROC curve analysis, the most useful candidate markers for predicting KFD-MAS were absolute lymphocyte count and serum LDH level (Table 4).

**Table 4:** Receiver operating characteristic curve analysis of serum markers for KFD-MAS diagnosis

	AUC	Cut-off Value	Sensitivity (%)	Specificity (%)	95%CI	*p*-value
Lymophocyte count (/μL)	0.84	< 1235	80	79.1	0.74–0.95	< 0.0001
Platelet count (× 10^6^/μL)	0.76	< 171	60	90.7	0.61–0.90	0.003
Albumin (g/L)	0.76	< 35.6	66.7	86	0.59–0.92	0.01
LDH (IU/mL)	0.99	> 679	86.7	97.7	0.97–1.0	< 0.0001

*AUC* area under the receiver operating characteristic curve, *KFD* Kikuchi-Fujimoto disease, *MAS* macrophage activation syndrome, *LDH* lactate dehydrogenase

## Discussion

In this study, as the previously reported incidence (30.8%) of MAS in KFD adults, a total of 25.9% of our patients presented with KFD-MAS [9]. The occurrence may be a bit overestimated since some mild cases without referral to our hospital might be missed. KFD tends to have female predominance with a female-to-male ratio of 1:3 in young patients of ages 15–18 years [10]. However, as some reported pediatric series [10–12], we found a greater proportion of males with KFD, especially in KFD-MAS patients with a female-to-male ratio of 1:2.75.

Most clinical manifestations between patients with KFD with and without MAS were not significantly different but there was a greater proportion of skin rash in our KFD-MAS patients. Similarly, despite no statistical significance, a previous report has found that a higher ratio of KFD-MAS patients tended to present with skin rash [9]. Therefore, there might be an association between skin rash and MAS in KFD, and future studies are required to confirm this interesting finding.

Compared to KFD patients without MAS, more KFD-MAS patients required glucocorticoid treatment. Corticosteroids and IVIG can be considered effective treatment options for KFD with and without MAS; etoposide could be an alternative regimen for severe KFD-MAS. Unlike the reported adult patients with KFD-MAS, our series showed no deaths. Compared to patients aged < 50 years, those aged 50 years and older had significantly higher mortality in KFD with MAS [9]. These findings suggest a possible better prognosis in younger patients, especially in the pediatric population with KFD-MAS.

Similar to most previous studies, our series had a noted higher recurrence rate of 15.5% than the reported recurrence rate of 3–4% in adults [11, 13, 14]. The higher recurrence rates in the pediatric population may be related to the difference in the immune status due to age and a possible higher diagnosis rate in those patients. Compared to KFD patients without MAS, our series showed a significantly higher recurrence rate in KFD-MAS patients. Previous studies have reported that leukopenia and lymphopenia were significantly associated with KFD recurrence [15, 16]. Thus, more severe leukopenia and lymphopenia may partially account for the higher recurrence rate in KFD-MAS patients.

A comparison of the laboratory findings showed a lower absolute peripheral white blood cell, platelet, serum albumin levels, lymphocyte count, and higher LDH levels in KFD-MAS patients. Further ROC curve analysis for the KFD-MAS diagnosis revealed that serum LDH level > 679 IU/mL had the highest areas under the ROC curve, and an absolute lymphocyte count < 1235/μL was the second. The diagnostic criteria for MAS in SJIA used in this study have not been validated for KFD. Clinical and laboratory characteristics of the patients, particularly the trend observed in the platelet count, levels of ferritin, transaminases, and fibrinogen, should still be the main elements in the early diagnosis of HLH in children. From these findings in this study, an extremely elevated serum LDH level and moderate to severe lymphopenia could help to find more patients with KFD-MAS.

The limitations of this study are the small sample size in a single center and the that it is retrospective. More than half of recurrences were diagnosed clinically without repeat lymph node biopsies. Bone marrow evaluation and serum TG levels were not detected in all patients. Future prospective studies with large sample sizes may help to further explore laboratory markers for the KFD-MAS diagnosis.

## Conclusions

The presence of KFD-MAS in children may be more common than previously expected, especially in those with skin rash. It may be associated with a higher recurrence rate. Severe lymphopenia may be a helpful predictor for KFD recurrence. An extremely elevated serum LDH level and moderate to severe lymphopenia might be useful diagnostic markers for MAS in KFD.

## Supplementary Information

**Additional file 1.**

## Data Availability

The datasets were collected from the medical records of participating patients at Shenzhen Children’s Hospital.

## Abbreviations

KFD: Kikuchi-Fujimoto disease
SLE: Systemic lupus erythematosus
JIA: Juvenile idiopathic arthritis
MAS: Macrophage activation syndrome
AST: Aspartate aminotransferase
ESR: Erythrocyte sedimentation rate
CRP: C-reactive protein
LDH: Lactate dehydrogenase
IVIG: Intravenous immunoglobulin
IQR: Interquartile ranges
ROC: Receiver operating characteristic curve
